# Daily blood feeding rhythms of laboratory-reared North American *Culex pipiens*

**DOI:** 10.1186/1740-3391-12-1

**Published:** 2014-01-22

**Authors:** Megan L Fritz, Edward D Walker, Aaron J Yunker, Ian Dworkin

**Affiliations:** 1 Department of Entomology, North Carolina State University, Raleigh, NC 27607, USA; 2 Department of Microbiology and Molecular Genetics, Michigan State University, East Lansing, MI 48824, USA; 3 Department of Zoology, Michigan State University, East Lansing, MI 48824, USA

**Keywords:** Blood feeding, *Culex pipiens*, Molestus ancestry, Plasticity

## Abstract

**Background:**

Blood feeding by free-living insect vectors of disease is rhythmic and can be used to predict when infectious bites will occur. These daily rhythms can also be targeted by control measures, as in insecticide-treated nets. *Culex pipiens* form pipiens and *C.p.* f. molestus are two members of the *Culex pipiens* assemblage and vectors of West Nile Virus throughout North America. Although *Culex* species vector human pathogens and parasites, the daily blood feeding rhythms of *C.p.* f. molestus, to our knowledge, have not been studied. We described and compared the daily blood feeding rhythms of three laboratory-reared populations of *Culex pipiens*, one of which has confirmed molestus ancestry. We also examined the plasticity of blood feeding time for these three populations.

**Results:**

For most (>70%) *C.p.* f. pipiens and *C.p.* f. molestus collected from metropolitan Chicago, IL, blood feeding took place during scotophase. Peak blood feeding occurred in mid-scotophase, 3-6 hours after lights off. For *C.p.* f. pipiens originating from Pennsylvania, most mosquitoes (> 90%) blood fed during late photophase and early scotophase. *C.p.* f. molestus denied a blood meal during scotophase were less likely to blood feed during early photophase (< 20%) than were *C.p.* f. pipiens from Chicago (> 50%). *C.p.* f. pipiens from Pennsylvania were capable of feeding readily at any hour of photo- or scotophase.

**Conclusions:**

Daily blood feeding rhythms of *C.p.* f. molestus are similar to those of *C.p.* f. pipiens, particularly when populations originate from the same geographic region. However, the timing of blood feeding is more flexible for *C.p*. f. pipiens populations relative to *C.p.* f. molestus.

## Background

Feeding is one of many insect activities governed by a circadian clock [1]. Insects feed and move in rhythmic patterns, punctuated by intervals of inactivity or sleep [2]. Endogenous feeding rhythms are entrained to daily light/dark cycles, although a number of other external and internal factors (*i.e*. temperature, wind, physiological state, etc.) can modulate these feeding rhythms [1]. In insects, adult feeding is restricted to light [3,4], dark [5-7], or dawn/dusk periods [6,8,9], whereas immatures may feed indiscriminately throughout the 24 hour day [10]. Across all life stages, divergence of feeding activity periods can be found among closely-related insect species, or within species [6,8-13].

Daily feeding rhythms of blood feeding insects [6,7,13-16] are well-studied, particularly in vector species, due to their implications for disease transmission. Successful blood meal acquisition by free-living blood-feeders (*i.e.* mosquitoes, biting midges, sandflies, etc.) requires that their active periods overlap with periods of host availability [17]. Yet predator activity, and environmental conditions (*i.e.* temperature, humidity) also have predictable 24 hour cycles, and can influence insect fitness [17]. The circadian clock of blood-feeding insects provides a mechanism whereby the timing of host-seeking can be synchronized with 1) host availability, 2) predator inactivity, and 3) suitable environmental conditions. For example, peak feeding by the bedbug, *Cimex lectularius*, occurs in the early morning hours, when human hosts are inactive and available for feeding, yet relative humidity is high [17]. Most pathogen and parasite transmission by disease vectors occurs during peak feeding periods. Awareness of peak blood feeding times by vector species, as well as their molecular basis, is useful for the development of control measures that reduce contact between blood feeding insects and their hosts. Details of the endogenous molecular clock underlying daily behavioral rhythms in some vector species have recently been described [18,19].

Among mosquitoes, foraging flights are rhythmic with respect to photoperiod [20-23]. Host cues also stimulate host finding and feeding behavior [24], however. One of these host cues, CO_2_, is thought to activate mosquito blood foraging [reviewed in 16], and serves as a long-range cue for host-finding [25]. For a number of mosquito species, the presence of nearby host cues, including CO_2_, may alter the timing of foraging behavior [16]. Yet laboratory studies of the role of CO_2_ at close range suggest that the importance of CO_2_ relative to other host cues varies by species [16].

Here, we describe the daily blood-feeding rhythms of three populations of *Culex pipiens.* One population of *Culex pipiens* form pipiens (hereafter *C.p.* f. pipiens) was originally collected in Pennsylvania. The other two populations, one *C.p*. f. pipiens and one *Culex pipiens* f. molestus (hereafter *C.p.* f. molestus) originated from metropolitan Chicago. We quantified the frequency and plasticity of blood feeding behavior during a 24 hour period for these three populations under controlled laboratory conditions. We also examined the extent to which the presence of CO_2_ modulates daily blood feeding rhythms for these populations.

Mosquitoes in the *Culex pipiens* assemblage are cosmopolitan and vector the pathogens that cause avian malaria, Bancroftian filariasis, St. Louis encephalitis, and West Nile Fever [26,27]. In North America, this assemblage includes *C.p.* f. pipiens, *C.p.* f. molestus, and *C. quinquefasciatus*[27]. Two bioforms, *C.p.* f. pipiens and *C.p.* f. molestus, are morphologically identical, have overlapping geographic distributions, but are behaviorally distinct. For example, *C.p.* f. molestus can oviposit their first batch of eggs prior to blood feeding, whereas a blood meal is obligatory for reproduction in *C.p.* f. pipiens [28]. *C.p.* f. molestus populations often breed underground (*i.e.* cellars, subway tunnels) [29-31], whereas *C.p.* f. pipiens populations breed above ground. Daily flight patterns of *C.p.* f. molestus from Asia are arrhythmic relative to other above-ground breeding *C. pipiens* assemblage members [23,32]. Their dark habitat with inconsistent light sources and evidence for flight activity arrhythmia suggest that *C.p.* f. molestus are less likely to be entrained by light as a zeitgeber compared with above-ground *C.p.* f. pipiens. Wild populations of these two bioforms are also reported to have distinct host preferences: *C.p.* f. pipiens specializes on avian hosts [33,34], whereas *C.p.* f. molestus feed on humans [30,35]. Because of their propensity to bite humans, the *C.p.* f. molestus may serve as an important bridge vector for West Nile virus in North America [36]. Yet to our knowledge, no studies have described the daily blood feeding rhythms, and peak feeding times of the molestus bioform.

We predicted that peak feeding times of *C.p.* f. molestus and *C.p.* f. pipiens would differ based on the following:

1) Peak flight activity of inseminated females is correlated with peak feeding time [16], and *C.p.* f. molestus flight activity lack a daily rhythm relative to above ground breeding *C. pipiens*, [23,32]*.*

2) Females of these two bioforms are reported to prefer different host species, which may be inactive (*ie*. available to serve as blood hosts [19]) at different times during the day.

We found that the majority of feeding took place in the dark for all populations, which is consistent with studies of other *Culex* species [6,37]. For both *C.p.* f. pipiens and *C.p.* f. molestus populations from metropolitan Chicago, peak feeding occurred 3–6 hours after lights off. Neither the presence of CO_2_, nor the availability of a non-preferred blood type influenced peak feeding times. When deprived of a blood meal during scotophase, *C.p.* f. pipiens was significantly more likely to feed during daylight hours than was *C.p.* f. molestus. While peak feeding times of *C.p.* f. pipiens and *C.p.* f. molestus were similar, *C.p.* f. pipiens was more flexible with respect to blood feeding time than was *C.p.* f. molestus.

## Methods

### Mosquito rearing

**Figure 1:**
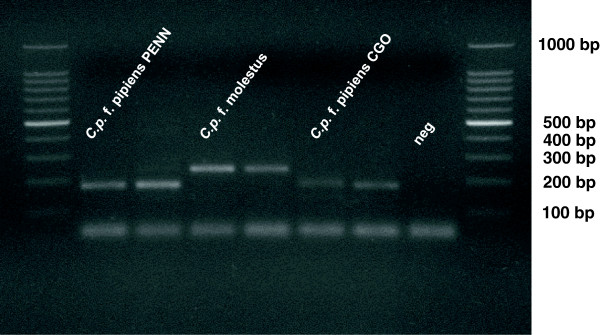
**Example agarose gel image confirming the ancestry of the 3 study populations of *C. pipiens *according to Bahnck and Fonseca **[36]**.** Amplicons from individuals in our study populations are flanked by a DNA ladder ranging from 1000 to 100 bp. From left to right, central bands represent individuals sampled from our *C.p.* f. pipiens PENN (n = 2), *C.p.* f. molestus (n = 2), *C.p.* f. pipiens CGO (n = 2) populations, with a negative control.

A *C.p.* f. molestus (Forskål) colony originating from an underground population in Calumet, IL [31] was acquired in April 2009 from the Centers for Disease Control (Fort Collins, CO). Molestus ancestry was indicated by > 90% oviposition prior to blood feeding (Fritz *et al.* unpublished) [28], and confirmed by PCR assay [38] (Figure 1). *C.p.* f. pipiens (Linnaeus), originating from Pennsylvania (hereafter PENN), was acquired in January 2011 from a long-standing colony reared by the New York State Health Department. In August 2011, eggs collected from above ground gravid traps in Wolfe Wildlife Refuge, Oak Lawn, IL were used to initiate a second *C.p.* f. pipiens colony (hereafter CGO). Individuals from this colony were anautogenous (*i.e*. no autogenously produced egg rafts were observed), and their pipiens ancestry was confirmed by PCR assay (Figure 1).

Adults of each population were reared in 60 × 60 × 60 cm white BugDorm-2 insect cages (Megaview Science Education Services Co., Taiwan). Ten percent sucrose solution and a dark-bottomed dish (12 cm diameter) containing non-chlorinated tap water were provided *ad libitum*. Cages were held in an environmental chamber maintained at 28 ± 1°C and 50 ± 10% relative humidity under an LD 15:9 h photoperiod without dawn/dusk transitions. In their natural environment, insects are exposed to low light levels during scotophase (*i.e.* moonlight). Therefore, we used a night light, illuminated by a 4-W tungsten bulb to provide indirect, low light (0.17 lux) to mosquito cages during scotophase. This practice is important for laboratory studies of circadian behavior, because night-active insects may not behave normally in complete darkness [1].

To sustain colony egg production, blood was offered to adult mosquitoes via artificial membrane feeder [39] twice per week for at least one hour either shortly before or shortly after lights off. Blood feeding time was rotated by colony and date, and colonies were not always offered a blood meal at the same time each day. Populations were reared on blood of host classes that reflect their preferences in the wild [34]. The *C.p.* f. molestus colony was offered Na-heparinated horse blood (Hemostat Laboratories, Dixon, CA), while the *C.p.* f. pipiens colonies were offered Na-heparinated goose blood (Lampire Biological Laboratories, Pipersville PA) sweetened with a 50% sucrose solution according to Moudy *et al.*[40]. All colonies were blood fed through pork sausage casing (Great Lakes Butcher Supply Co., Howell, MI).

Eggs and larvae were collected from ovipositional dishes twice per week and were transferred to 27 × 19.4 × 9.5 cm plastic pans containing 700–800 ml of tap water. Pans were thinned to contain no more than 250 larvae, and were fed 6 ml of 1% (v/v) liver powder and brewer’s yeast solution (2:1 parts desiccated liver powder:brewer’s yeast Sigma-Aldrich Co. St. Louis, MO) every other day. Larvae and pupae were exposed to fluorescent light levels of 700 lux (Lumichrome, 40 W, 122 cm T12, 5000 K, CRI96, M & M Lighting Co., Ronan, MT, U.S.A) during photophase, and < 0.17 lux during scotophase. Pupae were picked from rearing pans with a plastic Pasteur pipette, and transferred to a 12 cm diameter plastic dish inside its corresponding adult rearing cage daily.

Experiments began in July 2011 and were conducted under the environmental conditions used for rearing. Upon eclosion, adult males and females of each population were held together and allowed to mate for 5 days. One important difference between *C.p.* f. pipiens and *C.p.* f. molestus is that *C.p.* f. molestus postpones blood feeding until completion of their first egg-laying bout [28]. For our *C.p.* f. molestus, first oviposition occurs between the first 4–7 days of adult life [Fritz *et al*., unpublished]. We provided female *C.p.* f. molestus additional time to oviposit; female *C.p.* f. pipiens and *C.p.* f. molestus were one and two weeks old respectively at the time of testing. Thus the timing of readiness for blood feeding was similar for *C.p.* f. molestus and *C.p.* f. pipiens despite a difference in ages. Twenty-four hours prior to testing, 6 day old female *C.p.* f. pipiens, and 13 day old *C.p.* f. molestus were transferred to clean 30 × 30 × 30 cm white BugDorm-1 insect rearing cages (Mega View Science Education Services Co., Taiwan) for testing. During mating and ovipositional periods, all mosquitoes were provided 10% sucrose solution *ad libitum*. Water, but not sucrose solution, was provided *ad libitum* during testing.

### Experiment 1 – single cage tests of blood feeding rhythm

Twenty-four hours prior to testing, 20–25 adult female *C.p.* f. pipiens of the PENN and CGO populations and *C.p.* f. molestus were aspirated into each of three test cages. During testing, blood feeding via artificial membrane feeder was monitored over 24 hours. We divided the 24 hour test into 4 different periods, where hour 0 represents the start of the test: i.) late photophase (0–3 hours), ii.) scotophase (3–12 hours), iii.) early photophase (12–15 hours), and iv.) intermediate photophase (15–24 hours). Beginning in late photophase, mosquitoes were allowed access to the blood feeder, and the blood and membranes were replaced every three hours thereafter until intermediate photophase. Whenever the blood and membranes were changed, blood fed females were counted and removed from their cages via mouth aspirator. During scotophase, a Tactikka Plus Headlamp (Petzl, West Valley City, UT, USA) with a red light filter was used to improve visibility for mosquito removal and data collection. Based upon previous work with other *Culex* species, we expected blood feeding to be low during the 9 intermediate hours of photophase [6]. Therefore, we examined blood feeding during these hours with a single count of blood fed females at the end of the 9 hour period.

One of the three following treatments was applied to cages during testing:

1) In the absence of a constant CO_2_ source, *C.p.* f. pipiens of the CGO and PENN populations were offered Na-heparinated goose blood, while *C.p.* f. molestus were offered Na-heparinated horse blood. For the *C.p.* f. pipiens, and *C.p.* f. molestus populations, this was replicated 9 and 7 times, respectively.

2) To determine whether offering a non-preferred blood type influenced feeding rhythms, an additional 6 replicate groups of *C.p.* f. molestus and *C.p.* f. pipiens PENN were offered goose and horse blood, respectively. Here, a constant CO_2_ source was also absent.

3) To determine whether the presence of CO_2_ influenced feeding rhythms, 6 replicate groups of *C.p.* f. pipiens of the CGO and PENN populations were offered Na-heparinated goose blood, while 6 replicate groups of *C.p.* f. molestus were offered Na-heparinated horse blood in the presence of CO_2_. A 50 g block of dry ice was placed on the tops of the cages, adjacent to the blood feeder, and changed every three hours during testing. Under our testing conditions, the mean hourly CO_2_ release rate of a 50 g block of dry ice was 258 mL/min. This release rate was within the acceptable range for host attraction by both human- and avian-seeking mosquito species [41].

### Experiment 2 – multi-cage tests of blood-feeding rhythm in the absence of CO_2_

To examine feeding time plasticity, 7 separate Bugdorm-1 cages each held 15–25 mosquitoes of a single strain. During a 24 hour test period, each of the 7 cages was offered a blood meal at a different three hour feeding interval. Thus, some individuals were offered a blood meal during their preferred feeding window, and for others, feeding was advanced or delayed. As in Experiment 1, blood feeding began in late photophase and continued for 24 hours. The blood and membranes were replaced every three hours. Blood fed females from each cage were counted upon removal of the blood feeder.

### Statistical analysis

All statistical analyses were conducted using R version 2.15.1 [42]. Minimal adequate models were fit by sequentially eliminating model terms using likelihood ratio parametric bootstrap tests (number of simulations = 100). Model terms were retained if the p-value from the comparison of full and reduced models was less than 0.05. We confirmed the results of our likelihood ratio tests by comparing full and reduced models using Akaike’s Information Criterion (AIC).

For Experiment 1, we examined whether mosquito population, time, and their interaction influenced the proportion of mosquitoes that fed. Responses (proportion fed) by treatment group 1 were modeled over time using mixed logistic regression with a binomial error distribution (lme4) [43]. To account for the autocorrelation between measurements taken on a single cage across time, we fit a random effect that allowed the proportion fed from each cage to vary by three hour interval. We reported results from the following full model to examine blood feeding response by the *i*th cage:

$$\begin{array}{l} A.\;\mathrm{Pr}\;\left[y_{i}=1\right]=\log it^{-1}(\beta_{0i}+\beta_{1}\;\mathrm{Tim}e_{i}+\beta_{2}\;\mathrm{Populatio}n_{i}\\ \;+\beta_{{}_{1}}\beta_{{}_{2}}\;\mathrm{Population}:\mathrm{Tim}e_{i}+u_{0i}+u_{1i}\mathrm{Tim}e_{i}) \end{array}$$

for *i* = 1,…, n

where *u*_*0*_ ~ N(0, σ^2^_*0i*_) and *u*_*1*_ ~ N(0, σ^2^_*1i*_), represents the random effect of cage with a covariance estimated between them.

To determine whether the blood of different host species influenced feeding behavior, we used logistic regression to model the time until feeding in response to population, treatment and their interaction. Individuals who fed in the first three hour interval were scored as having an elapsed feeding time of 0 (interval (I) = 0), and individuals feeding in any subsequent three hour intervals (I = 1 – 5) were given an elapsed feeding time of (I × 3). Responses by *C.p*. f. molestus and *C.p*. f. pipiens PENN individuals from treatment groups 1 and 2 were fit using the glm function from the stats package [42] where residuals were specified as gamma distributed. Treatment comparisons were not made for *C.p.* f. pipiens CGO because the population had not been collected when testing began. We reported results from the following full model, that examines time to feeding by the *i*th individual:

$$\begin{array}{l} B.y_{i}=\log it^{‒1}\;(\beta_{0i}+\beta_{1}\;\mathrm{Populatio}n_{i}+\beta_{2}\;\mathrm{Blood}\;\mathrm{Treatmen}t_{i}\\ \;+\beta_{1}\beta_{2}\;\mathrm{Population}:\mathrm{Blood}\;\mathrm{Treatmen}t_{i}) \end{array}$$

for *i* = 1,…, n

We also used logistic regression to model the effects of population, CO_2_ and their interaction on elapsed time until feeding by the *i*th mosquito, via the stats package as above. Residuals were specified as gamma distributed for the following full model:

$$\begin{array}{l} C.y_{i}=\log it^{-1}\;(\beta_{0i}+\beta_{1}\;\mathrm{Populatio}n_{i}+\beta_{2}CO_{2i}\\ \;+\beta_{1}\beta_{2}\;\mathrm{Population}:CO_{2i}) \end{array}$$

for *i* = 1,…, n

For Experiment 2, mosquito responses were modeled using mixed logistic regression with a binomial error distribution. We used the following full model to determine whether population, time, or their interaction influenced the feeding by the *i*th mosquito at each three hour interval:

$$\begin{array}{l} D.\mathrm{Pr}\;\left[y_{i}=1\right]=\log it^{-1}(\beta_{0i}+\beta_{1}\;\mathrm{Populatio}n_{i}+\beta_{2}\;\mathrm{Tim}e_{i}\\ \;+\beta_{1}\beta_{2}\;\mathrm{Population}:\mathrm{Tim}e_{i}+u_{0i}) \end{array}$$

for *i* = 1,…, n

where *u*_*0*_ ~ N (0, σ^2^_*i*_) represents the random effect of test date.

## Results

### Experiment 1 – single cage tests of blood feeding rhythm

**Figure 2:**
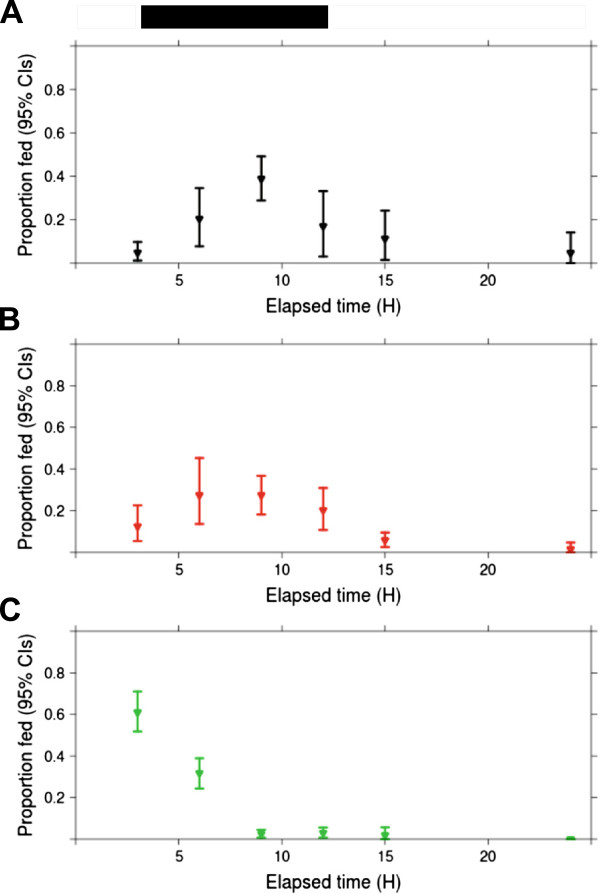
**The observed proportion of individuals fed (± bootstrapped 95% CIs) at each time interval in the absence of CO**_**2**_**.** Panels **A-C** represent blood feeding rhythms by single cages of *C.p*. f. molestus (black), *C.p.* f. pipiens CGO (red), and *C.p*. f. pipiens PENN (green), respectively. Bars above panel A represent the light cycle (white = photophase, dark = scotophase).

In the absence of CO_2_, the proportion of fed mosquitoes differed with population and time of day (p < 0.001). Most *C.p*. f. pipiens PENN fed in the late hours of photophase, shortly after testing was initiated (Figure 2). Their mean (2.5%, 97.5% CIs) elapsed time to feeding was 1.51 (1.17, 1.86). Therefore, most *C.p.* f. pipiens PENN fed within the first three hours of testing (I = 0). In contrast, most *C.p.* f. pipiens CGO and *C.p.* f. molestus fed during scotophase (Figure 2). Mean elapsed times to feeding for *C.p*. f. pipiens CGO and *C.p.* f. molestus were 5.2 (4.7, 5.7), and 6.9 (5.7, 7.5) hours, respectively.

Time to feeding changed little when *C.p*. f. molestus and *C.p.* f. pipiens PENN were offered non-preferred host blood. Mean time to feeding was 9.09 (8.25, 10.01) hours for *C.p.* f. molestus fed goose blood, and 1.49 (1.15, 1.85) hours for *C.p.* f. pipiens PENN fed horse blood. A comparison of the mean times to feeding (± 95% CIs) for *C.p*. f. molestus fed either goose, or horse blood suggested that offering a non-preferred blood type resulted in a modest delay of feeding. However, model reduction by likelihood ratio parametric bootstrap demonstrated that neither the interaction between blood treatment and mosquito population (p = 0.78), nor the blood treatment itself (p = 0.33) influenced time to feeding for *C.p.* f. molestus and *C.p.* f. pipiens PENN. For each host blood treatment, the observed percentage fed (± bootstrapped 95% CIs) per population across time are reported in Table 1.

**Table 1:** **Percentage of population fed for *C.p*. f. molestus and *C.p. *** **f. pipiens PENN when fed preferred vs. non-preferred blood types [following 28, 31, 33]**

	**N**				
**Strain**	**Total fed horse blood**	**Total fed goose blood**	**Elapsed time (h)**	**Horse blood**	**Goose blood**
*C.p.* f. molestus	88	92	3	***4.9 (1.1, 9.7)***	0 (0,0)
			6	***20.4 (7.4, 34.3)***	11.8 (3.7, 20.7)
			9	***38.9 (28.4, 49.0)***	24.3 (8.5, 42.7)
			12	***17.0 (3.3, 34.0)***	27.0 (20.3, 34.8(
			15	***11.3 (1.1, 23.9)***	28.8 (15.5, 42.0)
			>24	***7.7 (0, 14.1)***	7.9 (0, 12.5)
*C.p.* f. pipiens PENN	197	115	3	57.1 (41.0, 75.7)	***61.0 (52.0, 71.3)***
			6	38.7 (20.0, 56.0)	***31.8 (24.3, 39.0)***
			9	2.5 (0, 5.8)	***2.4 (0.7, 4.4)***
			12	1.6 (0, 5.0)	***3.0 (0.7, 5.6)***
			15	0 (0, 0)	***0.02 (0, 0.1)***
			>24	0 (0, 0)	***0 (0, 0)***

Preferred blood types for each population are in italics. Ninety-five percent confidence intervals were calculated via parametric bootstrapping (N = 5000).

**Figure 3:**
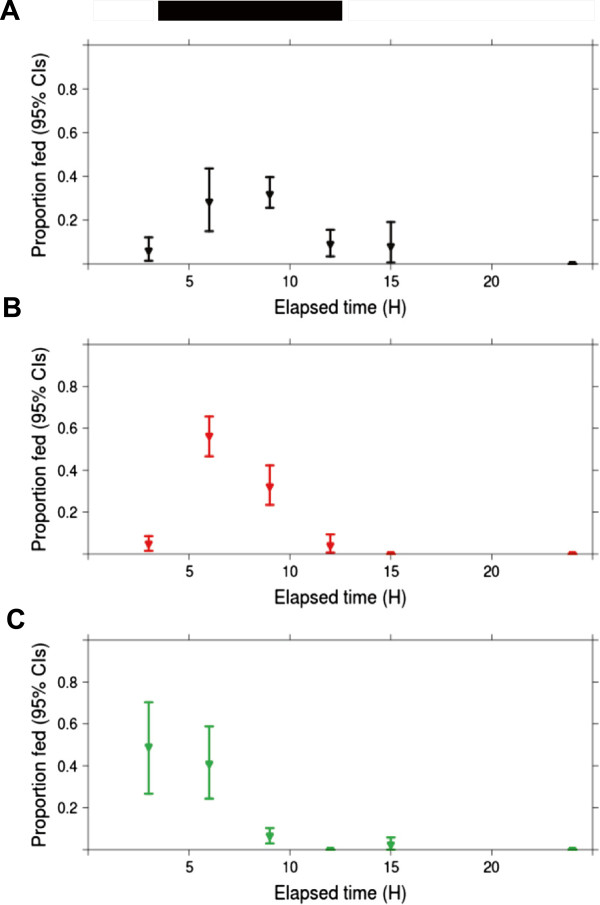
**The observed proportion of individuals fed (± bootstrapped 95% CIs) at each time interval in the presence of CO**_**2**_**.** Panels **A-C** represent blood feeding rhythms by single cages of *C.p*. f. molestus (black), *C.p.* f. pipiens CGO (red), and *C.p*. f. pipiens PENN (green), respectively. Bars above panel A represent the light cycle (white = photophase, dark = scotophase).

When CO_2_ was provided as a blood feeding cue, a greater proportion of the *C.p.* f. pipiens CGO blood fed in the early hours of scotophase (Figure 3). The *C.p.* f. pipiens CGO mean elapsed time to feeding was modestly shortened from 5.2 (4.7, 5.7) hours when CO_2_ was absent to 4.1 (3.8, 4.4) hours when CO_2_ was present. However, their median elapsed time to feeding in the presence of CO_2_ was 3 (3,3) hours, and not different from their median time to feeding in the absence of CO_2_. Time to feeding was not different for *C.p.* f. pipiens PENN and *C.p.* f. molestus by either measure of central tendency (Table 2). Likelihood ratio tests confirmed that including CO_2_ as a blood feeding cue did not influence elapsed time to feeding (p = 0.24). A summary of the mean and median elapsed times to feeding (± 95% CIs) for each population and treatment from Experiment 1 is provided in Table 2.

**Table 2:** Mean and median elapsed times until feeding for each population and treatment combination from Experiment 1

		**Elapsed time to feeding in hours (2.5%, 97.5% CIs)**	
**Treatment**	**Population**	**Mean**	**Median**
Preferred blood type without CO_2_	*C.p.* f. pipiens PENN	1.5 (1.2, 1.9)	0 (0, 0)
	*C.p.* f. pipiens CGO	5.2 (4.7, 5.7)	6 (3, 6)
	*C.p.* f. molestus	6.7 (5.9, 7.5)	6 (6, 6)
Non-preferred blood type without CO_2_	*C.p.* f. pipiens PENN	1.5 (1.2, 1.9)	0 (0, 3)
	*C.p.* f. molestus	9.1 (8.3, 10.0)	9 (9, 9)
Preferred blood type with CO_2_	*C.p.* f. pipiens PENN	1.8 (1.4, 2.2)	0 (0, 3)
	*C.p.* f. pipiens CGO	4.1 (3.8, 4.4)	3 (3, 3)
	*C.p.* f. molestus	5.3 (4.7, 5.9)	6 (3, 6)

Ninety-five percent confidence intervals were calculated via parametric bootstrapping (N = 5000).

### Experiment 2 – multi-cage tests of blood-feeding rhythm in the absence of CO_2_

**Figure 4:**
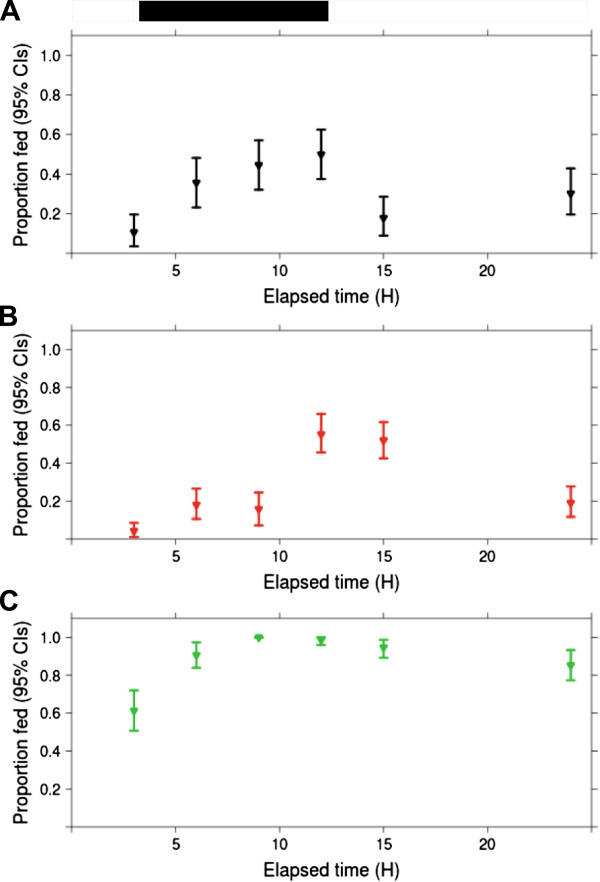
**Observed percentages fed by *C.p*. f. molestus (N = 336; in black), *C.p*. f. pipiens CGO (N = 539; in red), and *C.p*. f. pipiens PENN (N = 450; in green) over the 24 h day are represented by Panels A-C, respectively.** At each elapsed time interval, single cages of *ca.* 25 females from each population were applied to the blood feeder, and blood fed females were counted at the end of the 3 h interval. Bars above panel A represent the light cycle (white = photophase, dark = scotophase).

In Experiment 2, blood feeding depended interactively on mosquito strain and time of day (p = 0.05, Figure 4). The proportion blood fed for *C.p.* f. pipiens PENN was high (> 0.80, Figure 4) during scotophase and most of photophase. *C.p.* f. pipiens CGO, and *C.p.* f. molestus feeding was low during late photophase (< 0.10, Figure 4), and peaked in late scotophase (> 0.50, Figure 4). However, a greater proportion of *C.p.* f. pipiens CGO fed in the early hours of photophase (0.52) than did *C.p.* f. molestus (0.18).

## Discussion

Here, we described and compared the blood feeding rhythms of *C.p*. f. molestus from metropolitan Chicago, to *C.p.* f. pipiens populations originating from metropolitan Chicago and Pennsylvania. Despite the many behavioral and physiological differences, blood feeding rhythms of *C.p.* f. pipiens CGO and *C.p*. f. molestus were similar. Where warmth and moisture were present, the majority of blood feeding took place during night-time hours, which is consistent with previous findings in other *Culex* species [6,37]. For both populations, feeding peaked 3–6 hours after the onset of scotophase. Interestingly, the presence of CO_2_ did not modify blood feeding behavior for these two populations. This is consistent with other findings that suggest CO_2_ is of minor importance in laboratory studies [16], though we make no claims as to its importance as a long range activator of host seeking in the field.

The behavior of *C.p*. f. pipiens CGO and *C.p.* f. molestus diverged when females were only offered a blood meal during one three hour period. Few *C.p.* f. molestus deprived of a blood meal throughout scotophase fed in early photophase, yet under these same conditions, *C.p.* f. pipiens fed readily in early photophase (Figure 4). Therefore, *C.p*. f. pipiens blood feeding rhythms were more plastic than those of *C.p.* f. molestus. We speculate that the relative importance of photoperiod in determining when mosquitoes blood feed differs amongst *C. pipiens* populations. Perhaps increased light levels inhibit blood feeding in *C.p.* f. molestus individuals, whereas this inhibition is diminished in *C.p.* f. pipiens. In the wild, blood feeding rhythm plasticity is likely important to *C.p.* f. pipiens because blood feeding is required for reproduction. Maintaining the flexibility to obtain a blood meal whenever hosts become available may enhance reproductive fitness.

The more restricted feeding rhythms of *C.p*. f. molestus may reflect historic patterns of human availability. It is interesting that the human-seeking *Culex* form and the highly specialized African malaria mosquito, *Anopheles gambiae*, have similar peak feeding times [44]. Widespread distribution of insecticide-treated bed nets (ITNs) have recently uncoupled typical night time feeding patterns of *A. gambiae* from human host availability throughout sub-Saraharan Africa [45]. Some *A. gambiae* populations have reportedly shifted their peak blood feeding time [46,47]. In North America, where housing is less permeable to mosquitoes, we question why this same selective pressure to shift feeding times does not appear to have affected *C.p*. f. molestus. Perhaps selection for feeding rhythm plasticity is weaker for autogenous forms like *C.p*. f. molestus because most individuals are capable of reproduction before a blood meal. Alternatively, the host range of North American *C.p.* f. molestus populations may be more flexible than those from European populations [30] which may also diminish the selection pressure to shift host feeding times.

One alternative explanation for the differences in plasticity by *C.p.* f. pipiens and *C.p.* f. molestus is their difference in age at the time of testing. We designed our experiment to minimize the differences between bioforms according to their readiness for blood feeding (see Methods). By necessity, this design led to a one week difference in age for the two bioforms used for testing. Aging dampens expression of circadian clock genes [48] as well as circadian rhythmicity of physiological processes and behaviors [49]. It is possible that the age differences between *C.p.* f. pipiens and *C.p.* f. molestus females used in the study contributed to the observed differences in behavior. Evidence from *A. aegypti* and *C. tarsalis* suggests this is unlikely, however. Other host-seeking traits, including host choice [24], flight activity [50], and flight distance [51] are modified little, if at all, by small age differences. Further research directed at understanding the effect of individual mosquito ages on the daily feeding rhythms of mosquito populations would enhance our ability to target and control vector species.

To our knowledge, we are the first to document the blood feeding rhythms of North American *C.p*. f. molestus. Studying the behavior of *C.p.* f. molestus in the field is challenging because populations are typically small and occupy environments that are difficult for researchers to access. Laboratory studies of rhythmic behavior in captive mosquito populations often reflect those of their wild counterparts [16], and here we show that laboratory studies of captive *C.p.* f. molestus are tenable. While we cannot rule out the possibility that captivity has influenced the blood feeding rhythms seen in our *C.p.* f. molestus population, this seems unlikely. Laboratory rearing of mosquitoes appears to increase behavioral plasticity rather than diminish it [52].

Blood feeding rhythms of *C.p*. f. pipiens PENN not only differed from *C.p.* f. molestus, but also from *C.p.* f. pipiens CGO. Average time to feeding was shorter for *C.p*. f. pipiens PENN relative to other populations, and blood feeding peaked in late photophase (Figure 2). Yet when blood feeding was restricted to one three hour interval, any hour of scotophase, or early photophase appeared equally good for blood feeding (Figure 4). Indeed, when a blood meal was offered to *C.p.* f. pipiens PENN during daylight hours, > 60% of the population blood fed. In contrast, blood feeding by *C.p*. f. molestus was rare during daylight hours (Figure 4). This suggests that the timing of blood feeding by *C.p.* f. pipiens PENN is extraordinarily flexible. It is unclear whether this plasticity reflects the true phenotype of the ancestral population collected from Pennsylvania, or whether it is the result of colonization for over a decade. However, other field-collected *Culex* species have flexible blood feeding rhythms, with a likely genetic basis [11, Severson personal communication].

## Conclusions

The blood feeding rhythms of *C.p.* f. molestus and *C.p.* f. pipiens are similar for populations collected from the same geographic region. However, when deprived of a blood meal, *C.p*. f. pipiens populations are more likely to feed during the early morning hours than are *C.p*. f. molestus. This suggests that the timing of blood feeding is much more flexible for *C.p*. f. pipiens and more restricted for *C.p.* f. molestus.

## Availability of supporting data

The data sets supporting the results of this article are available in the Dryad repository http://doi.org/10.5061/dryad.jf1bv.

## Competing interests

The authors declare that they have no competing interests.

## Author’s contributions

MLF designed the experiments, analyzed the data, and wrote the manuscript. EDW participated in the experimental design and edited the manuscript. AJY participated in the experimental design, and collected the data. ID participated in the experimental design and data analysis, and edited the manuscript. All authors read and approved the final manuscript.
